# Factors affecting prepubertal and pubertal bone age progression

**DOI:** 10.3389/fendo.2022.967711

**Published:** 2022-08-22

**Authors:** Mari Satoh, Yukihiro Hasegawa

**Affiliations:** ^1^ Department of Pediatrics, Toho University Omori Medical Center, Tokyo, Japan; ^2^ Division of Endocrinology and Metabolism, Tokyo Metropolitan Children’s Medical Center, Tokyo, Japan

**Keywords:** bone age, endochondral ossification, growth plate, estrogen, hypogonadism, Turner syndrome

## Abstract

Bone age (BA) is a clinical marker of bone maturation which indicates the developmental stage of endochondral ossification at the epiphysis and the growth plate. Hormones that promote the endochondral ossification process include growth hormone, insulin-like growth factor-1, thyroid hormone, estrogens, and androgens. In particular, estrogens are essential for growth plate fusion and closure in both sexes. Bone maturation in female children is more advanced than in male children of all ages. The promotion of bone maturation seen in females before the onset of puberty is thought to be an effect of estrogen because estrogen levels are higher in females than in males before puberty. Sex hormones are essential for bone maturation during puberty. Since females have their pubertal onset about two years earlier than males, bone maturation in females is more advanced than in males during puberty. In the present study, we aimed to review the factors affecting prepubertal and pubertal BA progression, BA progression in children with hypogonadism, and bone maturation and deformities in children with Turner syndrome.

## What is bone age?

### Mechanism of bone maturation in the long bones

The long bones grow as a result of endochondral ossification, which contributes to height acquisition during childhood (1). In endochondral ossification, cartilage first develops and is later replaced by bone. There are two ossification centers in endochondral ossification, the primary and secondary centers. The primary ossification center finally forms the diaphysis of the long bones, whereas the secondary center forms the epiphysis. These two formations each proceed in basically the same way (2). The growth plate is located between the end of diaphysis (metaphysis) and the epiphysis, and its ossification is also brought about by endochondral ossification. Endochondral ossification at the growth plate is essential for the elongation of the long bones.

**Figure 1:**
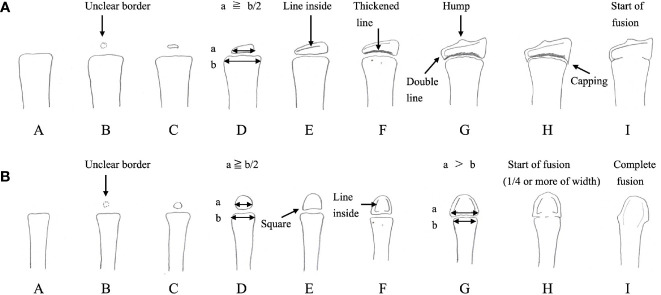
Bone maturation process and points of bone age evaluation using the Tanner-Whitehouse 2 method for the radius **(A)** and the third metacarpal bone **(B)** The epiphysis gradually enlarges, and its width becomes equal to, or greater than, that of the metaphysis. Afterwards, fusion of the growth plate begins, culminating in the complete fusion of the epiphysis and metaphysis. In the Tanner-Whitehouse 2 method, bone maturation is classified into stages A–H or I (3). The stage of bone maturation is determined by the size of the epiphysis, its shape and structure, and the degree of growth plate fusion. (Modified versions of Figure 6-21 and Figure 6-28 from Murata M et al., Assessment of skeletal maturity: A practical manual. Tokyo: HBJ (1997). 65 p. and 68 p.).

The term bone maturation denotes the developmental stage of endochondral ossification at the epiphysis and the growth plate for which bone age (BA) serves as a clinical marker. BA is evaluated by the size and shape of the epiphysis, which reflects the process of secondary ossification and the degree of fusion in the growth plate (3). **Figure 1** shows a graded scale of bone maturation in the radius and the third metacarpal bone according to Tanner-Whitehouse 2 (TW2) method (3).

An increase in height is the result of endochondral ossification; chondrocyte differentiation is followed by the replacement of cartilage with bone at the growth plate of the long bones (1). In short, chondrocyte progenitors in the resting zone move proximally to differentiate into proliferating and hypertrophic chondrocytes, which secrete extracellular matrix components. Subsequently, in the hypertrophic zone invasion of blood vessels leads to apoptosis of the chondrocytes and osteoblast migration and finally to bone formation in the matrix at the distal end of the metaphysis.

**Figure 2:**
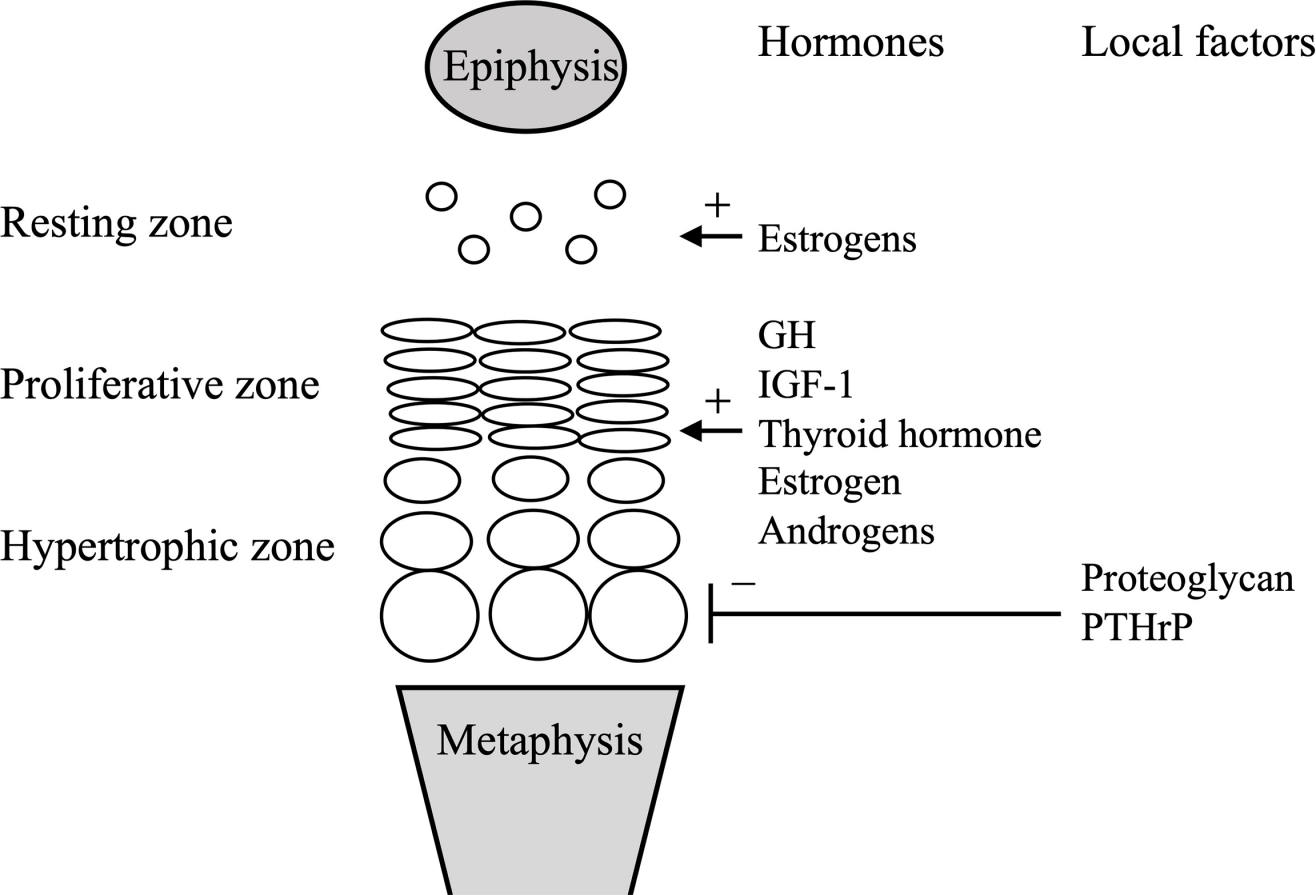
Simplified scheme of the growth plate. Systemic and local factors described in the manuscript are only shown. GH, growth hormone; IGF-1, insulin-like growth hormone-1; PTHrP, parathyroid hormone-related peptide.

Hormones promoting endochondral ossification include growth hormone (GH), insulin-like growth factor-1 (IGF-1), thyroid hormone, estrogens, and androgens (4). Children with GH deficiency and hypothyroidism show growth disturbance and BA delay, while children with precocious puberty and hyperthyroidism show growth promotion and BA acceleration (4). Estrogens are essential for the growth plate closure, a late-stage marker of BA development. In male patients with estrogen resistance and aromatase deficiency, the growth plates do not fuse, and height continues to increase even in adulthood (5, 6), whereas in female patients with Kallmann syndrome with isolated hypogonadotropic hypogonadism, the growth plates do not fuse even by age 20 years (7). Estrogens reportedly cause irreversible depletion of chondrocyte progenitors in the resting zone at the growth plate (8). **Figure 2** illustrates major factors that regulate the growth plate.

On the other hand, 46,XY patients with complete androgen insensitivity syndrome reached almost normal final height for females without treatment, suggesting that androgen is not essential for epiphyseal fusion (9). In patients with androgen insensitivity syndrome, estrogens converted from androgens promote bone maturation.

### Bone age assessment methods

The Greulich-Pyle (GP) (10) and TW2 (3) methods are used world-wide. The GP method was developed using radiographs of upper-middle-class Caucasian children in the United States collected between 1931 and 1942. The TW2 method was created using radiographs of average socioeconomic class children in the United Kingdom collected in the 1950s and 1960s. The GP method is atlas-based, whereas the TW2 method is a score and more objective. However, the TW2 method requires more time to evaluate than the GP method. The TW3 was corrected for the secular trend in bone maturation and published in 2001 (11).

In the TW2 method, the maturity level of each bone is classified into one of the stages from A to H or I according to the size and shape of the epiphysis and the degree of growth plate fusion. Each stage is assigned a score, and the total score is calculated to determine the BA. In the various version of the TW2 method, the conversion into the BA accounts for variations found in the target population of the respective region (12–14).

Several automated BA assessment methods have been developed, including the BoneXpert method (15). The BoneXpert method is a method in which the borders of 13 RUS bones (radius, ulna, and 11 short bones in fingers 1, 3, and 5) are automatically determined from a digitized image to calculate an intrinsic BA. This intrinsic BA is transformed into the GP bone age or TW bone age. This system’s usefulness is reported in various ethnic groups by comparing it to the other standards for each ethnic group (16, 17).

## Bone age before the pubertal onset

### Estrogens and bone maturation

Evaluation of left hand and wrist radiographs in male and female subjects demonstrates that bone maturation in healthy female children begins accelerating earlier than in healthy male children (3, 10). Bone maturation in female children may accelerate even before puberty, probably under the influence of estrogens, which ultrasensitive bioassays show are higher in female than in male children before puberty (18, 19); circulating estradiol is thought to be synthesized in the ovaries from adrenal steroids through FSH-induced aromatase upregulation before adrenarche (20).

### Adrenarche and the bone maturation

Adrenal androgens are presumably involved in bone maturation before pubertal onset. Adrenal androgens are produced in the adrenal cortex and have a weak androgen effect. The main adrenal androgens are dehydroepiandrosterone (DHEA) and DHEA-sulfate (DHEA-S), which act by being converted to more potent androgens, such as testosterone, and to estrogens in peripheral tissues (21).

Several pieces of circumstantial evidence argue for the effects of adrenal androgens on BA. First, delayed BA before pubertal onset in a case of complete adrenal androgen deficiency suggests that adrenal androgens are involved in prepubertal bone maturation, as was apparently the case in a Japanese female patient with the 46,XY karyotype with 17α-hydroxylase deficiency, whose BA at the chronological age (CA) of 10 years was 6 years for a female, and 7 years for a male, child (22).

Second, BA is advanced in children with premature adrenarche, which is caused by early adrenal maturation and is the most common cause of premature pubarche. In general, adrenarche involves the maturation of the adrenal zona reticularis. Biochemical adrenarche, which involves an increase in DHEA and DHEA-S, begins at age 5–6 years in both sexes (23). In children with premature adrenarche, BA before pubertal ages is significantly advanced in both sexes; one-third of children with this condition have an average BA, another one-third have a BA advanced by 1 to 2 years, and the remaining one-third have a BA advanced by more than 2 years (24).

Third, in children born small for gestational age (SGA), BA in early childhood lags the CA but catches up to near equivalency at pubertal onset (25). Serum DHEA-S and androstenedione levels in male and female children at the age eight years were inversely related to birth weight and birth length (26), and serum DHEA-S was found to be higher in short, female children born SGA than children in a control group with a CA of 6.5 to 7 years (27), indicating that early adrenal maturation onset may be responsible for the acceleration of the bone maturation in short, prepubertal children born SGA.

Finally, obese, prepubertal children reportedly have accelerated linear growth accompanied by advanced BA. Pubertal height gain is smaller in obese children than in non-obese children because BA at pubertal onset in the former is advanced (28). Increased DHEA-S was also found to contribute to the advanced BA in obese, prepubertal children (29). Furthermore, as these children have large amounts of adipose tissue where aromatase is expressed, the increase in estrogens converted from adrenal androgens may promote earlier bone maturation (30).

### Local factors in the growth plate

Several local factors, such as the so-called cartilage matrix factors, are involved in endochondral ossification of the growth plate and bone maturation in addition to hormones. First, aggrecan, encoded by *ACAN*, is a proteoglycan component in the extracellular matrix of the growth plate and articular cartilage. Heterozygous *ACAN* mutations lead to short stature with variable phenotypes, such as idiopathic short stature with advanced BA, spondyloepiphyseal dysplasia, and spondyloepimetaphyseal dysplasia (31, 32). Advanced BA before pubertal onset is characteristic of patients with *ACAN* mutations. Premature hypertrophic chondrocyte maturation, early growth of blood vessels, and osteoblast migration into the hypertrophic zone are thought to underlie advanced, prepubertal BA in patients with *ACAN* mutations (33, 34) on the basis of the findings of a study using chicks (34).

Other rare clinical disorders manifesting advanced, prepubertal BA related to proteoglycan are Desbuquois dysplasia type 1, which is caused by inactivating mutations of *CANT1* (35), and Desbuquois dysplasia type 2, which is caused by activating mutations of *XYLT1* (36). In these disorders, impairment of proteoglycan synthesis leads to short stature with advanced BA, as seen in patients with *ACAN* mutations.

Finally, patients with a decrease in the alpha subunit of G protein (Gsα)-cAMP-protein kinase A (PKA) pathway signaling, as seen in Albright’s hereditary osteodystrophy, acrodysostosis type1, caused by inactivating mutations of *PRKAR1A*, and acrodysostosis type 2, caused by activating mutations of *PDE4D*, also exhibit short stature with an advanced BA (37). Specifically, decreased Gsα-cAMP-PKA pathway signaling causes advanced BA associated with decreased parathyroid hormone-related peptide (PTHrP) signaling at the growth plate, which accelerates the normal differentiation process of growth plate chondrocytes (38).

## Bone age during puberty

### Sex hormones and bone maturation

Sex hormones are essential for bone maturation during puberty. Since females have their pubertal onset about two years earlier than males, bone maturation in females is more advanced than in males during puberty.

In female and male patients with hypogonadotropic hypogonadism, BA is delayed from adolescence to adulthood. In female patients, this delay is indicative of the importance of the role of estrogens, and in male patients, of the role of androgens and estrogens converted from androgens in the peripheral tissues and growth plate cartilage. A 17-year-old male patient with isolated hypogonadotropic hypogonadism caused by a GnRH receptor (*GNRHR*) mutation had no pubertal development and a BA of 14.5 years, and his 16-year-old sister with the same mutation had no thelarche and a BA of 12.5 years (39). A 20-year-old female Japanese patient with Kallmann syndrome had poor breast development and a BA of 13 years (7), and a 22-year-old male Brazilian patient with Kallmann syndrome caused by a *KAL1* mutation had 3mL testes and a BA of 14 years (40).

Delayed BA from adolescence to adulthood in patients of both sexes with aromatase deficiency indicates that estrogens play a major role in bone maturation in both sexes. A 14-year-2-month-old female patient with aromatasedeficiency with no palpable breast tissue had a BA of 10 years (41) and a 14-year-7-month-old female Sri Lankan patient with the same condition had Tanner stage 1 breast development and a BA of 10.1 years (42). An adult male Caucasian patient had a BA of 15 years when he received the diagnosis of aromatase deficiency at age 28 years (6).

In the absence of sex hormones, bone maturation begins to decelerate at the BA equivalent to the mean CA at pubertal onset and does not progress beyond the BA equivalent just before the start of growth plate fusion. In male Japanese patients with GH deficiency associated with gonadotropin deficiency who received GH therapy, BA decelerated after age 12 years and did not progress beyond age 14 (43). Furthermore, in male Japanese children who had short stature at puberty and received GH and GnRH analog therapy, BA advancement began to slow after age 12 years and did not progress beyond age 14 years (44). Similarly, in female Japanese children with short stature receiving GH and a GnRH analog, BA deceleration occurred after age 10.5 years and did not progress beyond 12 age years (44). In untreated Japanese patients with Turner syndrome, the BA maturation decelerated after a BA of 10 years and never exceeded a BA of 12–13 years (45). The bone ages in all the cases mentioned above were evaluated using the TW2-radius, ulna, and short bones (RUS) method standardized for Japanese children (46).

The BA of 12 years for Japanese males and 10.5 years for Japanese females with decelerating bone maturation caused by the absence of sex hormones nearly match the mean CA at pubertal onset [testicular volume > 3 mL at age 11.5~12 years and breast development ≧ Tanner stage 2 at age 10 years (47)]. In addition, the BA of 14 years in Japanese males and 12 years in Japanese females correspond to the BA just before the start of growth plate fusion, suggesting that sex hormones are essential for growth plate fusion. Thus, sex hormones are necessary for bone maturation during adolescence, and estrogens are essential for growth plate fusion and closure in both sexes. The effect of estrogens on growth plate fusion is discussed in the following chapter.

### Estrogens and growth plate fusion

Estrogens have two roles in bone maturation. One is that they increase height by promoting growth plate chondrocytes’ differentiation, proliferation, and apoptosis, and the other is to promote growth plate fusion and closure. The latter processes require not only an irreversible depletion of chondrocyte progenitors in the resting zone but also senescence of the growth plate, which involves a decline in the growth and proliferation rates, number, and size of chondrocytes (48). Growth plate fusion is thought to be triggered when the proliferative potential of the growth plate chondrocytes is finally exhausted.

The duration between pubertal onset and growth cessation is longer in untreated children with precocious puberty than in children with normal puberty because BA at the pubertal onset in the former is lower than in normal children. Hypothetically, the growth plates in young children are less senescent and thus require prolonged estrogen exposure to complete the senescence process, which triggers growth plate fusion (48).

Other clinical evidence for growth plate senescence indicates that the growth velocity peaks at a BA of 13 years and 11 years in male and female Japanese children, respectively, before declining (49), possibly because of the senescence of the growth plate, i.e., the decline in the ability of chondrocytes to proliferate.

## Bone age in children with Turner syndrome

### Madelung deformity

The *short stature homeobox* (*SHOX*) gene is located on a short arm pseudoautosomal region of the X and Y chromosomes, and SHOX haploinsufficiency causes short stature and skeletal deformities (50). Léri-Weill dyschondrostenosis, a type of SHOX haploinsufficienc*y*, and some instances of Turner syndrome result in a short fourth metacarpal bone and radius and ulna deformities, collectively known as Madelung deformity, which is related to premature fusion of the lesions (50). This premature fusion, one form of bone maturation advancement, is probably caused by SHOX haploinsufficiency. Since the haploinsufficiency may be related to an aberrant cell death process in the growth plate (51), the overall BA is delayed as described below. However, bone maturation in the fourth metacarpal bone and the distal radioulnar regions advances.

Premature fusion of the growth plate in SHOX haploinsufficiency is conspicuous at the distal ends of the radius and ulna, presumably attributable to the high expression level of SHOX. In fact, at Carnegie development stage 14, SHOX is widely expressed near the middle of the limbs (52). Then, at Carnegie development stage 21, SHOX expression is localized in the upper limbs to the humerus, distal radius, ulna, the distal end of each bone in the lower limbs, and the first and second arches (52).

Because estrogens promote growth plate fusion, Madelung deformity in SHOX haploinsufficiency is accelerated during puberty and severer in female patients. The prevalence of Madelung deformity in Turner syndrome is relatively low at 7.5%, possibly owing to hypogonadism, a common complication of the disorder (50). In addition, patients with Turner syndrome receiving estrogen therapy from the late teens starting at a low dosage rarely have Madelung deformity (53).

### Bone maturation before and during puberty in children with Turner syndrome

The mean ΔBA/ΔCA in untreated Japanese patients with Turner syndrome with no spontaneous puberty was 0.75 ± 0.63 before BA 10 years (54), owing to chromosome imbalance, hypogonadism, and SHOX haploinsufficiency.

SHOX is expressed in the growth plate, particularly in hypertrophic chondrocytes, and regulates chondrocyte differentiation, hypertrophy, and apoptosis (55). When chondrocyte apoptosis is suppressed by SHOX haploinsufficiency, bone maturation is delayed owing to the delay in the replacement of chondrocytes by osteoblasts. BA lags the CA in prepubertal patients with SHOX haploinsufficiency, although the delay is not very significant. In prepubertal male and female patients with *SHOX* variants and SHOX upstream or downstream enhancer deletions, the mean BA/CA was 0.9 ± 0.1 (56). In prepubertal, male and female patients with SHOX haploinsufficiency, the mean difference between BA minus CA was -0.9 ± 0.9 (57). BA was evaluated using the GP method, but because the data were analyzed without regard to sex, it is unclear whether a sex difference in bone maturation was present before pubertal onset in these patients.

BA advancement after puberty in SHOX haploinsufficiency is complicated because bone deformity in the hands may deteriorate with puberty (53), as discussed above. Madelung deformity, which involves earlier bone maturation in some of the bones in the hands (50), may be the combined manifestation of SHOX haploinsufficiency and estrogen exposure. Pubertal development in patients with SHOX haploinsufficiency is normal.

To investigate the extent to which hypogonadism and SHOX haploinsufficiency are each involved in bone maturation in prepubertal and pubertal patients with Turner syndrome, the degree of BA delay should be compared in patients with Turner syndrome with the 45,X karyotype and female patients with SHOX deletion.

## Conclusion

Estrogens are involved in prepubertal and pubertal BA progression and are essential for growth plate fusion and closure. Adrenal androgens are the source of estrogens before pubertal onset. In the absence of sex hormone, bone maturation begins to decelerate at the BA equivalent to the mean CA at the pubertal onset. It does not progress beyond the BA equivalent to just before the start of growth plate fusion. In addition to hormones, factors such as cartilage matrix and SHOX are also involved in bone maturation.

## Author contributions

YH planned the review. MS searched for previous articles and wrote the first draft of the manuscript. MS and YH reviewed and discussed the draft, and MS completed the manuscript submitted. All authors contributed to the article and approved the submitted version.

## Funding

This study received a grant from Japan Agency for Medical Research and Development (AMED 22ek01099464s0403) (YH).

## Acknowledgments

We are indebted to Mr. James R. Valera for his assistance with editing this manuscript.

## Conflict of interest

The authors declare that the research was conducted in theabsence of any commercial or financial relationships that couldbe construed as a potential conflict of interest.

## Publisher’s note

All claims expressed in this article are solely those of the authors and do not necessarily represent those of their affiliated organizations, or those of the publisher, the editors and the reviewers. Any product that may be evaluated in this article, or claim that may be made by its manufacturer, is not guaranteed or endorsed by the publisher.
